# Role of 3D Transesophageal Echocardiography for Transcatheter Mitral Valve Repair—A Mini Review

**DOI:** 10.3389/fcvm.2022.815304

**Published:** 2022-02-02

**Authors:** Kensuke Hirasawa, Masaki Izumo

**Affiliations:** ^1^Department of Cardiovascular Medicine, Tokyo Medical and Dental University, Tokyo, Japan; ^2^Division of Cardiology, Department of Internal Medicine, St. Marianna University School of Medicine, Kawasaki, Japan

**Keywords:** mitral regurgitation, transesophageal echocardiography, transcatheter mitral valve repair, MitraClip, 3D

## Abstract

Edge-to-edge transcatheter mitral valve repair (TMVr) using MitraClip has been evolving rapidly in patients with severe mitral regurgitation (MR) at high surgical risk or having contraindications for surgery. Three-dimensional (3D) echocardiography plays an important role in the management of severe MR. In particular, 3D transesophageal echocardiography (TEE) imaging allows the evaluation of MV geometry and quantification of MR severity with dedicated software. Real-time 3D TEE is also commonly used to guide TMVr and facilitate the procedure. Further development of 3D echocardiography may help achieve safer and more beneficial results. The following article summarizes the current knowledge and the future perspectives of 3D TEE in TMVr.

## Introduction

Mitral regurgitation (MR) is one of the most common valvular heart diseases in developed countries. In the Framingham Heart Study, MR was observed in 19% of the participants (1) and the clinical burden is getting increased with age (2). Thus, patients with severe MR have been associated with a higher surgical risk, and the need for less invasive therapies has been increased.

In the last few decades, transcatheter mitral valve repair (TMVr) has developed rapidly as a treatment option for patients with severe MR at higher surgical risk or having contraindications for surgical mitral valve (MV) intervention. MitraClip (Abbott Vascular, Santa Clara, CA) is the most commonly used edge-to-edge TMVr device in the world that mimics the surgical Alfieri stitch. Several randomized control studies have demonstrated the benefits of the device in various clinical settings. The Endovascular Valve Edge-to-Edge Repair Study (EVEREST) II trial (3) demonstrated a lower prevalence of major adverse events at 30-days after an MV procedure in patients treated with MitraClip compared to open-heart surgery. Subsequently, the Clinical Outcomes Assessment of the MitraClip Percutaneous Therapy for High Surgical Risk Patients (COAPT) trial (4) showed clinical benefits using the MitraClip system in patients with severe secondary MR. Based on these results, the current ACC/AHA guidelines recommended transcatheter MV repair for symptomatic primary MR with high or prohibitive surgical risk and symptomatic secondary MR (grade ≥3+) with a left ventricular ejection fraction (LVEF) of 20–50% and LV end-systolic diameter ≤ 70 mm despite maximally tolerated guideline-directed optimal medical therapy (5).

Echocardiography has long been a key imaging modality for the evaluation of valvular heart diseases. The identification of detailed anatomical characteristics of the MV is essential to understand the mechanisms of the diseases and to select an optimal timing and treatment, however, conventional two-dimensional (2D) echocardiography has substantial limitations in assessing the complex anatomy of the MV apparatus inherent to the technical methodology. One of the main limitations is that 2D echocardiography can only show one acquisition plane which sometimes leads to misunderstanding of the MV geometry. Recent developments in ultrasound devices allow the characterization of the complex anatomy of cardiac structures with great accuracy using three-dimensional (3D) echocardiographic images with great accuracy (6, 7). In particular, real-time 3D transesophageal echocardiography (TEE) plays an indispensable role in the assessment and management of TMVr (8, 9). Herein, we summarize the current knowledge about the utility of 3D TEE for the management of edge-to-edge TMVr using MitraClip.

## Pre-Procedural Evaluation of Mitral Regurgitation

MR is generally classified into two phenotypes; primary (organic) MR and secondary (functional) MR.

**Figure 1:**
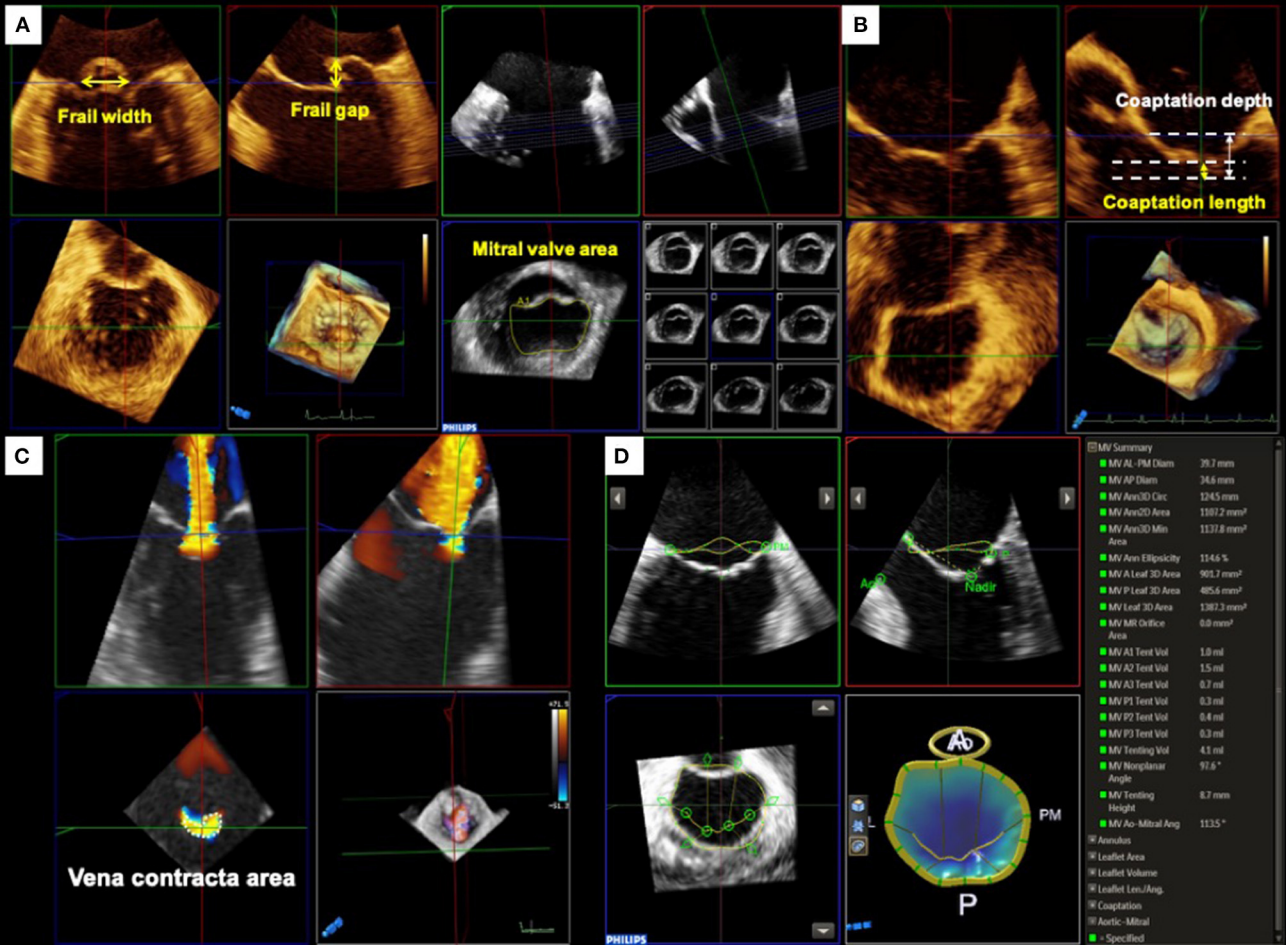
Pre-procedural assessment and quantification of mitral valve geometry. **(A)** Primary mitral regurgitation. For treating primary MR with MitraClip, the frail gap and width of the lesion and mitral valve opening area are used for assessing the procedural durability. **(B)** Secondary mitral regurgitation. Whereas, the coaptation depth and length should be evaluated for secondary MR. **(C)** Quantification of mitral regurgitation by three-dimensional (3D) color Doppler. Three-dimensional vena contracta area allows to evaluate regurgitant orifice area directly and may improve the assessment of regurgitant severity. **(D)** MV geometrical assessment using Mitral Valve Navigator^A.I.^. Semi-automated software dedicated to MV quantification provides useful information on the MV geometry from 3D TEE images.

Primary MR is characterized by degenerative alterations of the MV leaflet such as prolapse and/or frailty. Three-dimensional TEE allows to visualize the comprehensive anatomy of the MV and is helpful for easy understanding of the diseased lesion resulting from the degeneration. Moreover, a quantitative evaluation of MV anatomy must be performed to identify patients who will benefit from the procedure. In the EVEREST trials, several anatomical criteria for primary MR were used as follows; a frail gap <10 mm and a frail width <15 mm (10). In addition, several exclusion criteria, such as severe leaflet calcification in the grasping zone, leaflet perforation, significant cleft, and MV opening area <4 cm^2^, were defined. However, these measurements are sometimes difficult to assess using only conventional 2D images. Using 3D TEE with multiplanar reconstruction, a more accurate measurement of these dimensions can be obtained (Figure 1A).

Color Doppler 3D echocardiographic image is also informative for understanding the characteristics of MR. In many cases of primary MR, the eccentric direction of the regurgitant jet is commonly observed. Thus, it may be difficult to plan an optimal clip position. Color Doppler 3D TEE images depict the accurate location of the regurgitant orifice and the jet direction, which may help in planning the ideal positioning of the MitraClip.

In contrast, secondary MR is defined as MR due to LV and/or LA dysfunction without abnormalities in the MV leaflet and chordae tendineae (11). Although severe secondary MR is associated with adverse prognosis (12–14), the optimal treatment remains controversial. A recently published COAPT trial showed an incremental benefit of MitraClip implantation in addition to guideline-directed medical therapy in patients with symptomatic severe secondary MR at high surgical risk. In contrast, the Multicenter Study of Percutaneous Mitral Valve Repair MitraClip Device in Patients with Severe Secondary Mitral Regurgitation (MITRA-FR) trial showed no significant improvement in outcomes in patients treated with MitraClip. (15) The discrepancy in the results of these two randomized controlled trials may be due to the baseline characteristics of patients. Thus, the indications for TMVr therapy should be carefully evaluated (Figure 1B).

Quantitative assessment of MR severity is crucial for determining the indications for TMVr. However, quantitative assessment of secondary MR using 2D echocardiography has several limitations. In many cases with secondary MR, the flow convergence zone is not hemispherical and the regurgitant orifice has an oval or a crescent shape (16). Thus, the calculation derived by the proximal isovelocity surface area (PISA) method using 2D echocardiography can easily underestimate the MR severity (17, 18). Color Doppler 3D TEE and the multiplanar reconstruction provide a direct measurement of the regurgitant orifice area (3D VCA) which may improve the accuracy of MR grading (Figure 1C).

The MV geometry is also an important factor for considering the durability of TMVr. The COAPT trial used two anatomical inclusion criteria for secondary MR; coaptation length ≥2 mm and coaptation depth <11 mm. Several semi-automated echocardiographic software dedicated to 3D MV geometry have been introduced and applied for pre-procedural evaluation in clinical practice. MV area, perimeter, and leaflet area derived from 3D images can be measured using the software and may provide further incremental information about the degree of tenting and/or leaflet remodeling (19, 20) (Figure 1D).

Accordingly, the use of 3D TEE for selecting patients and evaluating the eligibility for TMVr is strongly recommended if available.

## Procedural Guidance of Mitraclip Using 3D Tee

During the TMVr procedure, TEE is generally used for guidance because interventionists require accurate geometrical information of the disease without direct inspection, unlike open-heart surgery. Clear visualization of the MV using 3D TEE images leads to better communication between imaging specialists and interventionists compared with 2D TEE. The utility of 3D TEE for procedural guidance has been demonstrated by a previous study that reported that 3D TEE reduced the procedural time compared to conventional 2D guidance alone (9). Thus, now the 3D TEE is mandatory for successful and safe TMVr therapy.

### Transseptal Puncture

**Figure 2:**
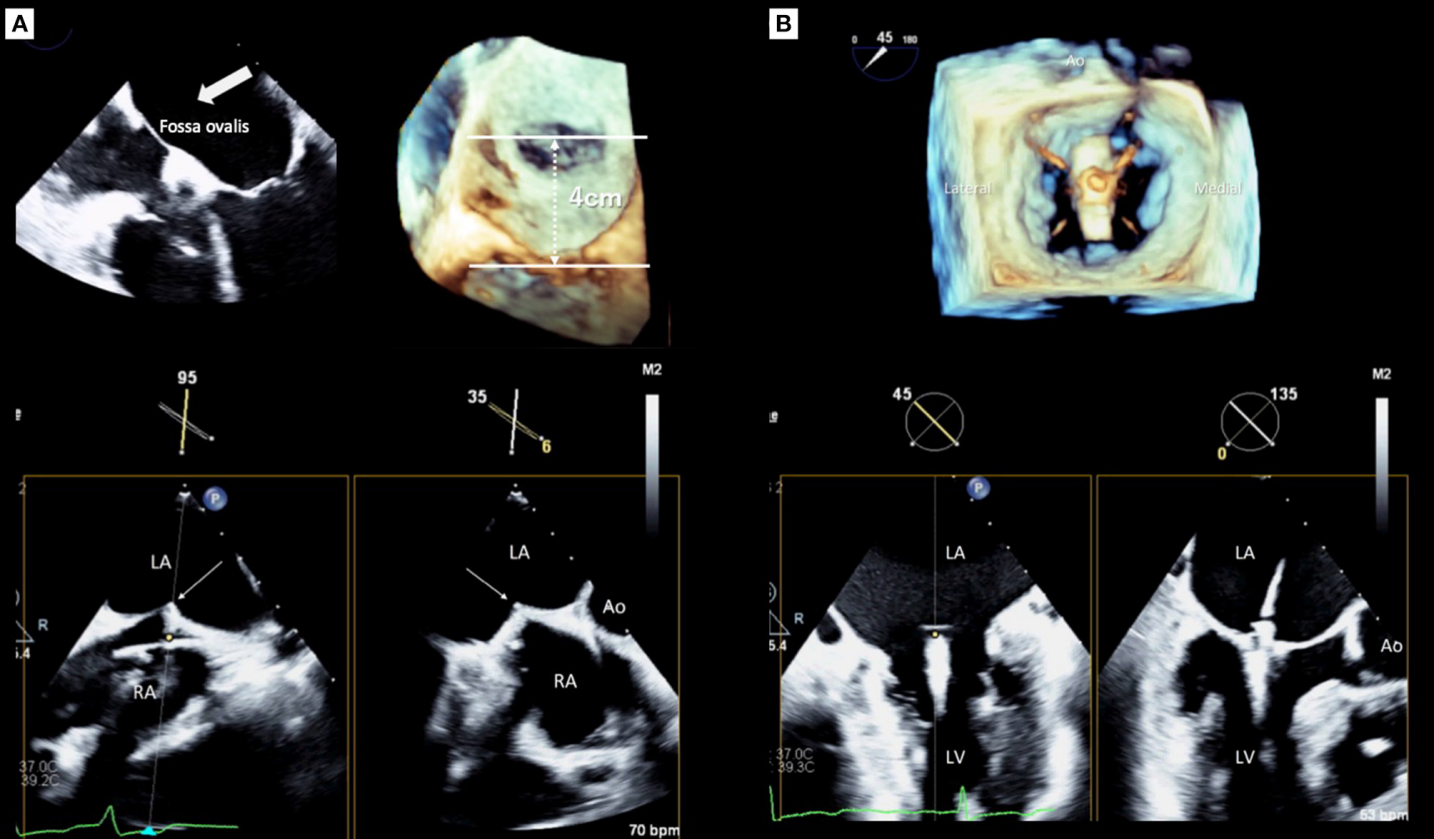
Real-time guidance for MitraClip implantation using three-dimensional transesophageal echocardiography. **(A)** Transseptal puncture guidance, **(B)** Clip deployment. LA, left atrium; LV, left ventricle; Ao, aorta.

Determining the optimal transseptal puncture site is an initial crucial role of the 3D TEE guide for TMVr because it fixes the position of the steerable guide catheter (SGC) and influences the mobility of the clip delivery system (CDS) (21). However, clear visualization of the targeted puncture site is sometimes difficult using only 2D images when the site is very posterior (22). Thus, a precise understanding of the interatrial septum and surrounding structures is required for a successful puncture, and the puncture site should be posterosuperior of the interatrial septum. The superior-inferior and the anterior-posterior coordinates of optimal puncture site are commonly confirmed using mid-esophageal bi-caval and short-axis views, respectively. Real-time 3D TEE can provide two planes simultaneously with the x-plane function and will facilitate the identification of the optimal position to be punctured (Figure 2A). However, the ideal puncture site was slightly different between the MR morphologies (23, 24). In patients with primary MR, the height of the puncture site has to be 4–5 cm from the mitral annulus. In contrast, in patients with secondary MR due to leaflet tethering, the height can be reduced because the leaflet coaptation plane shifts to the left ventricle. However, a height from the leaflet coaptation of <3.5 cm should be avoided because it may make the procedure difficult (24, 25).

### Guidance for Clip Deployment

After the CDS was inserted into the left atrium through the SGC, the clip was advanced into the LV. Two orthogonal echocardiographic views are normally used for the procedural guidance; an inter-commissural view and an LV outflow tract (LVOT) view from the mid-esophagus. The X-plane view provides these two planes simultaneously and enables the observation of the device. After the device was advanced to the LV using these X-plane views, the clip was slowly opened. Subsequently, the leaflets were grasped guided by TEE. Real-time 3D en-face MV view helps to assess the alignment of the clip which should be perpendicular to the line of the leaflet coaptation. After an initial grasp of the leaflets, the clip orientation must be evaluated (Figure 2B). Subsequently assessing the adequate insertion of both anterior/posterior leaflets, the MV geometry, which usually has a double-orifice, should be confirmed with the 3D en-face view. Before releasing the clip, the presence of residual MR has to be assessed carefully. Because patients treated with MitraClip had a higher prevalence of cardiac surgery during the first year after the procedure if significant residual MR exists in the EVEREST II trial (26). Moreover, significant residual MR has been shown as a strong determinant of poor outcomes after TMVr in several studies (27–30). Pulmonary venous flow patterns may provide useful information for determining the severity of residual MR indirectly (31). However, quantification of residual MR after MitraClip implantation has been challenging using 2D TEE. The proximal isovelocity surface area method, which is commonly used for evaluating native MR, is not feasible for residual MR after MitraClip implantation since the residual jet may have multiple and eccentric orifices. Color Doppler 3D TEE images help find and visualize the jet if it exists. In addition, 3D VCA may be a feasible and reliable method for quantification of residual MR after TMVr (32). If these results were acceptable, the clip was released. Finally, all evaluations must be performed again to compare the pre- and post-procedural results using both 2D and 3D TEE. If the clip location and the reduction of MR are not appropriate, a new attempt should be made to adjust the clip location.

## Future Perspectives and Discussion

Recent technological development will be clinically applied for the management of TMVr.

Dedicated applications of 3D echocardiography provide better visualization and more accurate quantification of MV anatomy than before.

Real-time fusion imaging of 3D echocardiography and fluoroscopy provides useful information for understanding the positional relationship between MV and the surrounding structures. It facilitates that both echocardiographers and interventionists share the same recognition of MV geometry, which may lead to a better post-procedural result.

In summary, physicians have required a better understanding and more accurate quantification of the MV geometry as TMVr has rapidly developed for patients with severe MR at high surgical risk. Real-time 3D TEE has become an indispensable and essential modality for the diagnosis and management of MR, and guidance during the TMVr procedure. With the appropriate use of 3D TEE, a more accurate assessment can be achieved in both primary and secondary MR. It also allows echocardiographers to share recognition of the MV geometry with interventionists and facilitates the procedure. Furthermore, the technological development of echocardiographic devices will allow a better illustration of complex anatomical MV morphology and may result in risk reduction after TMVr.

## Author Contributions

KH and MI drafted the manuscript. MI prepared the figures. All authors read and approved the final manuscript.

## Conflict of Interest

MI is a training faculty of Abbot Medical Japan. The remaining author declares that the research was conducted in the absence of any commercial or financial relationships that could be construed as a potential conflictof interest.

## Publisher's Note

All claims expressed in this article are solely those of the authors and do not necessarily represent those of their affiliated organizations, or those of the publisher, the editors and the reviewers. Any product that may be evaluated in this article, or claim that may be made by its manufacturer, is not guaranteed or endorsed by the publisher.

## References

[1] Abudiab MM Chao CJ Liu S Naqvi TZ. Quantitation of valve regurgitation severity by three-dimensional vena contracta area is superior to flow convergence method of quantitation on transesophageal echocardiography. Echocardiography. (2017) 34:992–1001. 10.1111/echo.13549 28480555

[2] Andell P Li X Martinsson A Andersson C Stagmo M Zöller B. Epidemiology of valvular heart disease in a Swedish nationwide hospital-based register study. Heart. (2017) 103:1696–703. 10.1136/heartjnl-2016-310894 28432156 PMC5749343

[3] Asgar AW Mack MJ Stone GW. Secondary mitral regurgitation in heart failure: pathophysiology, prognosis, and therapeutic considerations. J Am Coll Cardiol. (2015) 65:1231–48. 10.1016/j.jacc.2015.02.009 25814231

[4] Biner S Perk G Kar S Rafique AM Slater J Shiota T. Utility of combined two-dimensional and three-dimensional transesophageal imaging for catheter-based mitral valve clip repair of mitral regurgitation. J Am Soc Echocardiogr. (2011) 24:611–7. 10.1016/j.echo.2011.02.005 21435839

[5] Buck T Eiswirth N Farah A Kahlert H Patsalis PC Kahlert P. Recurrence of functional versus organic mitral regurgitation after transcatheter mitral valve repair: implications from three-dimensional echocardiographic analysis of mitral valve geometry and left ventricular dilation for a point of no return. J Am Soc Echocardiogr. (2021) 34:744–56. 10.1016/j.echo.2021.02.017 33722676

[6] Bushari LI Reeder GS Eleid MF Chandrasekaran K Eriquez-Sarano M Rihal CS. Percutaneous transcatheter edge-to-edge mitraclip technique: a practical “step-by-step” 3-dimensional transesophageal echocardiography guide. Mayo Clin Proc. (2019) 94:89–102. 10.1016/j.mayocp.2018.10.007 30611459

[7] Buzzatti N De Bonis M Denti P Barili F Schiavi D Di Giannuario G. What is a “good” result after transcatheter mitral repair? Impact of 2+ residual mitral regurgitation. J Thorac Cardiovasc Surg. (2016) 151:88–96. 10.1016/j.jtcvs.2015.09.099 26545970

[8] Cheng R Dawkins S Tat E Makar M Hussaini A Makkar RR. Relation of residual mitral regurgitation despite elevated mitral gradients to risk of heart failure hospitalization after MitraClip repair. Am J Cardiol. (2017) 120:1595–600. 10.1016/j.amjcard.2017.07.027 29025679

[9] Feldman T Foster E Glower DD Kar S Rinaldi MJ Fail PS. Percutaneous repair or surgery for mitral regurgitation. N Engl J Med. (2011) 364:1395–406. 10.1056/NEJMoa1009355 21463154

[10] Feldman T Kar S Rinaldi M Fail P Hermiller J Smalling R. Percutaneous mitral repair with the MitraClip system: safety and midterm durability in the initial EVEREST (Endovascular Valve Edge-to-Edge REpair Study) cohort. J Am Coll Cardiol. (2009) 54:686–94. 10.1016/j.jacc.2009.03.077 19679246

[11] Goliasch G Bartko PE Pavo N Neuhold S Wurm R Mascherbauer J. Refining the prognostic impact of functional mitral regurgitation in chronic heart failure. Eur Heart J. (2018) 39:39–46. 10.1093/eurheartj/ehx402 29020337

[12] Hirasawa K Namazi F Milhorini Pio S Vo NM Ajmone Marsan N Bax JJ. Insufficient mitral leaflet remodeling in relation to annular dilation and risk of residual mitral regurgitation after MitraClip implantation. JACC Cardiovasc Imaging. (2021) 14:756–65. 10.1016/j.jcmg.2020.08.019 33129743

[13] Izumo M Shiota M Kar S Gurudevan SV Tolstrup K Siegel RJ. Comparison of real-time three-dimensional transesophageal echocardiography to two-dimensional transesophageal echocardiography for quantification of mitral valve prolapse in patients with severe mitral regurgitation. Am J Cardiol. (2013) 111:588–94. 10.1016/j.amjcard.2012.10.045 23206924

[14] Kaneko H Neuss M Weissenborn J Butter C. Impact of residual mitral regurgitation after MitraClip implantation. Int J Cardiol. (2017) 227:813–9. 10.1016/j.ijcard.2016.10.054 27823895

[15] Matsumura Y Fukuda S Tran H Greenberg NL Agler DA Wada N. Geometry of the proximal isovelocity surface area in mitral regurgitation by 3-dimensional color Doppler echocardiography: difference between functional mitral regurgitation and prolapse regurgitation. Am Heart J. (2008) 155:231–8. 10.1016/j.ahj.2007.09.002 18215591

[16] O'Gara PT Mack MJ. Secondary mitral regurgitation. N Engl J Med. (2020) 383:1458–67. 10.1056/NEJMcp1903331 33027570

[17] Obadia JF Messika-Zeitoun D Leurent G Iung B Bonnet G Piriou N. Percutaneous repair or medical treatment for secondary mitral regurgitation. N Engl J Med. (2018) 379:2297–306. 10.1056/NEJMoa1805374 30145927

[18] Otto CM Nishimura RA Bonow RO Carabello BA Erwin JP 3rd Gentile F. 2020 ACC/AHA guideline for the management of patients with valvular heart disease: A report of the American College of Cardiology/American Heart Association Joint Committee on Clinical Practice Guidelines. J Thorac Cardiovasc Surg. (2021) 162:e183–353. 10.1016/j.jtcvs.2021.04.002 33972115

[19] Paranskaya L D'Ancona G Bozdag-Turan I Akin I Kische S Turan GR. Residual mitral valve regurgitation after percutaneous mitral valve repair with the MitraClip® system is a risk factor for adverse one-year outcome. Catheter Cardiovasc Interv. (2013) 81:609–17. 10.1002/ccd.24586 22887450

[20] Paulsen JM Smith TW. Echocardiographic imaging of the mitral valve for transcatheter edge-to-edge repair. Interv Cardiol Clin. (2016) 5:17–31. 10.1016/j.iccl.2015.08.002 27852479

[21] Sannino A Smith RL 2nd Schiattarella GG Trimarco B Esposito G Grayburn PA. Survival and cardiovascular outcomes of patients with secondary mitral regurgitation: a systematic review and meta-analysis. JAMA Cardiol. (2017) 2:1130–39. 10.1001/jamacardio.2017.2976 28877291 PMC5710448

[22] Singh GD Smith TW Rogers JH. Targeted transseptal access for MitraClip percutaneous mitral valve repair. Interv Cardiol Clin. (2016) 5:55–69. 10.1016/j.iccl.2015.08.005 27852482

[23] Singh JP Evans JC Levy D Larson MG Freed LA Fuller DL. Prevalence and clinical determinants of mitral, tricuspid, and aortic regurgitation (the Framingham Heart Study). Am J Cardiol. (1999) 83:897–902. 10.1016/S0002-9149(98)01064-9 10190406

[24] Stone GW Lindenfeld J Abraham WT Kar S Lim DS Mishell JM. Transcatheter mitral-valve repair in patients with heart failure. N Engl J Med. (2018) 379:2307–18. 10.1056/NEJMoa1806640 30280640

[25] Sugeng L Shernan SK Salgo IS Weinert L Shook D Raman J. Live 3-dimensional transesophageal echocardiography initial experience using the fully-sampled matrix array probe. J Am Coll Cardiol. (2008) 52:446–9. 10.1016/j.jacc.2008.04.038 18672165

[26] Feldman T Kar S Elmariah S Smart SC Trento A Siegel RJ. Randomized comparison of percutaneous repair and surgery for mitral regurgitation: 5-year results of EVEREST II. J Am Coll Cardiol. (2015) 66:2844–54. 10.1016/j.jacc.2015.10.018 26718672

[27] Vahanian A Brochet E. Transseptal puncture for structural heart intervention: an old technique with new indications. Heart. (2017) 103:1830–7. 10.1136/heartjnl-2016-310483 28883033

[28] Wunderlich NC Siegel RJ. Peri-interventional echo assessment for the MitraClip procedure. Eur Heart J Cardiovasc Imaging. (2013) 14:935–49. 10.1093/ehjci/jet060 24062377

[29] Zamorano J Gonçalves A Lancellotti P Andersen KA González-Gómez A Monaghan M. The use of imaging in new transcatheter interventions: an EACVI review paper. Eur Heart J Cardiovasc Imaging. (2016) 17:835–5af. 10.1093/ehjci/jew043 27311822

[30] Zeng X Levine RA Hua L Morris EL Kang Y Flaherty M. Diagnostic value of vena contracta area in the quantification of mitral regurgitation severity by color Doppler 3D echocardiography. Circ Cardiovasc Imaging. (2011) 4:506–13. 10.1161/CIRCIMAGING.110.961649 21730026 PMC3224848

[31] Ikenaga H Yoshida J Hayashi A Nagaura T Yamaguchi S Rader F. Usefulness of intraprocedural pulmonary venous flow for predicting recurrent mitral regurgitation and clinical outcomes after percutaneous mitral valve repair with the MitraClip. JACC Cardiovasc Interv. (2019) 12:140–50. 10.1016/j.jcin.2018.09.034 30678793

[32] Avenatti E Mackensen B El-Tallawi KC Reisman M Gruye L Barker CM. Diagnostic value of 3-dimensional vena contracta area for the quantification of residual mitral regurgitation after MitraClip procedure. JACC Cardiovasc Interv. (2019) 12:582–591. 10.1016/j.jcin.2018.12.006 30826230

